# Unlocking human potential in the AI Age: how employee-AI collaboration transforms work engagement through dual psychological pathways

**DOI:** 10.3389/fpsyg.2025.1705671

**Published:** 2026-01-28

**Authors:** Lin Sun, Runsen Hu, Han Su

**Affiliations:** 1 Business School, Hohai University, Nanjing, China; 2 Surrey International Institute, Dongbei University of Finance and Economics, Dalian, China; 3 School of Management Science and Engineering, Anhui University of Finance and Economics, Bengbu, China

**Keywords:** creative self-efficacy, Employee–AI collaboration, JDR theory, meaningful work, work engagement

## Abstract

This research explores the impact of Employee–AI collaboration on work engagement, focusing on the psychological mechanisms involved. Given the increasing integration of artificial intelligence in workplaces, understanding these dynamics has become crucial for enhancing employee experience. Using a three-wave time-lagged design, data were collected from knowledge workers utilizing AI technologies in China, yielding 516 valid responses. The study employed confirmatory factor analysis, hierarchical regression, and structural equation modeling to analyze the data. The findings indicate that Employee–AI collaboration positively enhances work engagement by increasing employees' perceptions of meaningful work and creative self-efficacy, with meaningful work and creative self-efficacy serving as partial mediators in this relationship. This research contributes to the Job Demands-Resources theory by identifying Employee–AI collaboration as a novel job resource that fosters engagement through enhanced personal resources. The implications for AI system design and employee training highlight the importance of integrating AI into the workplace sensibly to maximize employee engagement and well-being.

## 1 Introduction

The blistering application of artificial intelligence (AI) to the work experience in organizations is fundamentally transforming the nature of work design, execution, and experience. In addition to enhancing efficiency and the quality of decisions, it is a fact that AI is increasingly working alongside the staff as a working partner, actively affecting the task execution, decision-making, and solving problems. Recent estimates indicate that an overwhelming percentage of work-related actions around the world is going to be affected by AI, and the cooperation of the employees with AI will be a prime basis of organizational competitiveness (McKinsey Global Institute, 2023).

Meanwhile, the implementation of AI in everyday labor presents radical psychological and interpersonal issues. AI can complement human abilities, but it could also stimulate the issues of job safety and job displacement as well, not to mention technostress, which highlights the necessity of analyzing not only the performance results but the psychological experience and wellbeing of employees working at work using AI (Raisch and Krakowski, 2021; Úbeda-García et al., 2025).

Out of these experiences, work engagement which has been defined as vigor, dedication, and absorption, has been given as one of the most obvious regards as central indicator of sustainable employee functioning and organizational effectiveness (Schaufeli et al., 2002; Rich et al., 2010). New developments refer to the idea that AI application could produce a mixed effect on work engagement: it can enhance engagement by making it more psychologically available and meaningful in the task, but also can destroy engagement with uncertainty and stress when implemented inadequately or irresponsibly (Liu and Li, 2025). New research also shows that human-AI cooperation should be of high quality to promote work engagement through the fulfillment of both the fundamental and developmental needs of employees (Wu et al., 2025).

Regardless of these developments, the current literature does not answer a number of important questions. First, the majority of the literature is devoted to the adoption of AI, its use, or immediate task results, providing few opportunities to understand the impact that long-term employee-AI interaction can have on the motivational process of employees and prolonged motivation to work. Second, the existing research is disjointed in the field of human-computer interaction, information systems, and organizational behavior, having no integrational theoretical base to describe how the collaboration between employees and AI shifts into fundamental work-related attitudes. Third, despite the growing calls to consider employee wellbeing when working in AI, the psychological processes in which the employee-AI collaboration should affect work engagement have not been studied enough, and the relationship can be described as a theoretical black box (Soulami et al., 2024).

In order to fill the above research gaps, the following research questions will guide the study:

RQ1: Does employee–AI collaboration enhance employees' work engagement?

RQ2: How do psychological mechanisms and key personal resources, such as meaningful work and creative self-efficacy, mediate the influence of employee–AI collaboration on work engagement?

With the solutions of these research questions, this study constructs a theoretically based model that connects employee–AI collaboration to work engagement based on specific psychological resource channels, this study uses the Job Demands Resources (JD-R) model to conceptualize employee-AI collaboration as a new work resource. JD R framework is specifically the right fit within this question as it explains the mechanism in which job resources activate the personal resources which in turn result in work engagement via a motivational channel. Based on this reasoning, we hypothesize that employee-AI interaction will boost work engagement by serving as the fortifier of two major personal resources meaningful work and creative self-efficacy. Meaningful work engages the perceptions of employees of having a purpose and importance on their work and creative self-efficacy indicates the level of their confidence in coming up with and applying new ideas, both of which play an important role in staying motivated and engaged in AI-enabled settings. Moreover, we also explore whether the employee to AI collaboration has a direct influence on work engagement outside of these mediating factors.

Based on analyzing a Chinese population of knowledge workers working actively with AI technologies, this work creates and evaluates a dual-path mediation model that integrates the role of employee-AI collaboration and work engagement. In such a way, we make three significant donations. To begin with, we expand the JD-R model, indicating employee-AI collaboration as one of the new job resources during the digital era. Second, we clarify the psychology behind the fact that employee-AI cooperation can increase the engagement in work using the mediating variables of meaningful work and creative self-efficacy. Third, presenting a combination of the knowledge in the human computer interaction and organization behavior, we provide a more detailed picture of how human-AI relationship influences the motivational process of employees in the workplace.

## 2 Literature review and research hypotheses

### 2.1 Theoretical foundations

According to the JD-R model (Bakker and Demerouti, 2017; Bakker, 2024; Demerouti et al., 2001), physical and mental health of the employees are influenced by the work environment as a result of job demands and job resources. Job demands refer to unremitting physiological, cognitive, or emotional loads needed at the workplace and are capable of causing health problems and exhaustion. Job resources are factors that enhance goal attainment, personal growth and development which give positive motivation. According to Scholze and Hecker (2024), the JD -R model offers an effective theoretical approach to comprehending digitization-induced changes in job demands and resources both by the presence of its positive (bright side) and negative (dark side) outcomes and the possibility to categorize these outcomes systematically.

The relationship between the dual path logic of the Job Demands-Resources (JD-R) model and the work engagement also has to be described before addressing our research model. JD-R model (Demerouti et al., 2001) originally proposed the model to explain the coexistence of health-impairment and motivational path within the workplace environment. According to this model, job demands can be the cause of stress and exhaustion, whereas job resources can motivate employees addressing their basic psychological needs, which should result in a better work engagement (Bakker and Demerouti, 2017). The former is experienced in the form of strain and burnout due to high or constant demand, and it decreases individual wellbeing and performance; the latter is compensation, which is based on meeting the fundamental psychological needs, arousing intrinsic involvement and Work Engagement, and consequently improving attitudes and performance (Schaufeli and Bakker, 2004; Christian et al., 2011). With the advancement of the research of the JD-R model, the scholars started paying attention to the significance of the personal resources of the framework. The researchers, (Xanthopoulou et al. 2007, 2009) suggested the personal resources, including self-efficacy and positive emotions are activated by job resources and are able to promote the work engagement that consequently enhances the performance of employees. This concept was extended by Crawford et al. (2010) who also stressed on the constant accumulation of personal resources in a resources-efficacy-engagement loops, which promotes favorable employee results.

As artificial intelligence (AI) becomes increasingly embedded in organizational processes, employee–AI collaboration has emerged as a novel and important job resource. According to Kong (2023), employee–AI collaboration refers to the interaction and cooperation between employees and AI systems during the work process. Employees leverage AI technology to enhance their work effectiveness, decision-making capabilities, and overall efficiency. This collaboration encompasses traditional task allocation and information exchange while also emphasizing ongoing interaction that fosters mutual adaptation and learning. In other words, the use of AI is instrumental and one-directional, whereas collaboration is an interactive, relationship-oriented process in which trust and mutual adaptation play a critical role (Kong, 2023). Relative to traditional resources, employee–AI collaboration is characterized by intelligence, context-awareness, and adaptability, facilitating the reshaping of task boundaries, the extension of individual capabilities, and increased efficiency in achieving goals (Jarrahi, 2018; Dellermann et al., 2019; Raisch and Krakowski, 2021). However, AI can also introduce technostressors—strain and exhaustion arising from constant connectivity, complexity, and uncertainty—that may adversely affect employee wellbeing (Tarafdar et al., 2019).

According to the recent empirical evidence, Chuang et al. (2025) employed three-wave statistics and Bayesian structural equations modeling that revealed that AI self-efficacy and generative AI use tend to improve the productivity and job satisfaction, approximately doubling the exhaustion and exacerbating the work-family conflict. In addition, a buffering effect of generative AI is also shown toward the disastrous effects of the technostress. These results are consistent with the two-way reasoning of the JD-R model, meaning that the studies on collaboration between employees and AI must examine its dual nature as a resource and demand. Consistent with our theoretical interest, we consider how employee-AI collaboration as a job resource can activate the motivational path with respect to personal resources to generate work engagement, in particular meaningful work and creative self-efficacy as personal resources. According to Kong (2023), such four employee-AI collaboration dimensions include task division, exchange of information, mutual adaptation and outcome of collaboration. Task division is reasonable division of tasks between employees and AI, which helps employees focus on creative and intricate problems and assign standardized and repetitious jobs to AI, thereby streamlining work processes and employee satisfaction and performance.

The exchange of information is viewed as the central point of collaboration between employees and AI, where employees would be able to obtain and understand the suggestions made by AI quicker, which will assist in knowledge sharing and provide a solid base of trust to work as a team, thus, enhancing performance at work and employee engagement. Mutual adaptation is focused on the continuous learning and adaptation of employees and AI to evolve with changing work conditions where AI has to rationalize its functions based on employee data to improve the human-machine collaboration. Last, collaboration results are concerned with the real impacts of employee-AI collaboration such as work performance, the ability to innovate, and employee satisfaction. The studies show that not only a high-quality collaboration improves short-term results of the approach, but it also plays a major role in the long-term career growth of the employees and in the work of the whole organization.

### 2.2 Employee–AI collaboration, work engagement

According to the Job Demands Resources (JD-R) model, collaboration between employees and AI can be represented as a new job resource that can address the goal achievement and help workers to cope with the work loads (Bakker and Demerouti, 2007, 2017). In the event that AI systems will work as co-operative partners, they will be able to raise efficiency in particular work, boost the quality of decisions, as well as reduce cognitive and emotional load related to a complicated work task and generate resource gains as one of the core aspects of the JD-R motivational process. This is likely to enhance increased levels of vigor, dedication, and absorption that are fundamental constituents of work engagement.

Furthermore, effective work with AI is also likely to bring employees timely feedback, informational support, and a sense of competence as other sources of advanced digital work support (Parker and Grote, 2022). The previous studies on the topic of human-machine collaboration and human-AI state that these supportive technologies can enhance the motivation of the employees toward their work as long as they are seen as empowering and having no control over the staff (Kellogg et al., 2020; Raisch and Krakowski, 2021). In this regard, the direct positive impact of employee-AI cooperation on the working engagement of employees is anticipated.

H1: Employee–AI collaboration is positively related to employees' work engagement.

### 2.3 Employee–AI collaboration, meaningful work

Earlier studies have already started to investigate the psychological basis of employee-AI partnership. As an example, (Yin 2024) based on the Protection Motivation Theory showed that the approach and avoidance motivation of employees influence how they engage in collaboration with AI, and leadership styles could assist employees in dealing with the perceived threats of AI use. These results emphasize the motivational richness of the employee-AI collaboration and reaffirm the necessity of integrative theoretical approaches to describe its downstream consequences. Meaningful work means subjective concern of employees to sense of purpose, value, and meaningfulness of their work (Rosso et al., 2010). Case of the complementary work of employees and intelligent systems in AI-enabled contexts restructures the task layouts and the form of the work experiences, which allow having a better understanding of how the work fits the organizational missions, customer needs, and value to the society. Replacing repetitive and routine elements, AI liberates the employees and allows them to focus more on the things that can be considered more complicated and more rational, changing the virus role of an executor to a problem solver/participant of the decision-making process. Role transitions attracting this level of perceiving task importance and increased professional responsibility tend to foster an internal sense of meaning (Kellogg et al., 2020; Parker and Grote, 2022). Also, the scope of impact and the volume of outputs of employees increase dramatically due to AI analysis and insight potentials; the further the employees reach a wider range of stakeholders, the more readily they will recognize their contributions to organizations and society, which continue to integrate Meaningful work (Rai et al., 2019; Bailey et al., 2019).

Simultaneously, the human human-machine interfaces with explainable goals and controllable systems through visualized feedback and context-aware suggestions will illuminate goal paths. Organizational members might get a better understanding of cause and effect relationship between performance and results and, as a consequence, work purpose and value (Amershi et al., 2019; Arrieta et al., 2019; Jarrahi, 2018). With a prolonged cooperation and continuous refreshment of the set of skills, the employees tend to be redefined as a professional identity, being not focused on the conventional roles but more on an AI partners/knowledge workers. The professional identification and pride also become solidified into the closed experience of meaning (Glikson and Woolley, 2020; Raisch and Krakowski, 2021).

H2: Employee–AI collaboration positively influences Meaningful work.

### 2.4 Employee–AI collaboration, creative self-efficacy

Creative Self-efficacy refers to the beliefs of persons in their ability to produce, champion and apply new ideas i.e., the stages between ideation and implementation, which are known as the core phases, and is intensely connoted with creative/innovative self-efficacy where the scales of the latter are used (Tierney and Farmer, 2002, 2011). The collaboration between employees and AI empowers creative self-efficacy in a number of important ways.

The first of them is that AI tools will offer performance scaffolding in the context of knowledge retrieval, problem generation, and quality assurance; thus, making the successful execution of tasks more predictable and frequent, collecting mastery experience, the strongest diagnostic efficacy cue (Amershi et al., 2019; Brynjolfsson et al., 2023).

Second, with the assistance of computational power and model potential, employees can solve complicated tasks that they could not solve in the past and see high-quality solutions, which can support the beliefs regarding their potential and growth opportunities (Parker and Grote, 2022; Dellermann et al., 2019).

Third, human-machine interaction is a learning process in itself, every visible capability development entails a positive store of competence-efficacy-engagement loop (Schuetz and Venkatesh, 2020; Llorens et al., 2007). Meanwhile, explainable AI increases perceived ability to control model logic and model outputs, decreasing uncertainty and powerlessness and further strengthening Creative Self-efficacy (Arrieta et al., 2019).

Findings in previous studies have shown that domain-specific efficiency, illustrated by creative/innovative self-efficacy, is a significant predictor of creative and innovation-related behaviors at the individual level (Tierney and Farmer, 2002, 2011; Hammond et al., 2011) and a relevant mediator of how contextual variables like leadership and learning orientation influence outcomes in innovation (Gong et al., 2009).

H3: Employee–AI collaboration positively influences Creative Self-efficacy.

### 2.5 The mediating role of meaningful work

The Jobs Demands Resources (JD-R) model reflects that job resources help to enhance work engagement by means of a motivational process where the personal resources of the employees are triggered (Bakker and Demerouti, 2007, 2017). Meaningful work is also one of the essential personal resources indicating that employees feel that their work has sense, meaning, and is related to their values.

According to previous studies, job resources (autonomy, feedback and task significance) that enable and support employees may contribute to the development of a more meaningful work, thereby leading to an increase in work engagement (Rosso et al., 2010; Steger et al., 2012). Employees who can feel they have a purpose behind their work tend to invest more of the cognitive, emotional, and physical energy into their jobs. Regarding the employee-AI collaboration, AI systems which can be considered as collaborative partners can boost the sense of purpose of employees by facilitating better decision-making, eliminating unneeded strain, and letting employees to concentrate on the work aspects of higher value. Based on the motivational pathway suggested by the JD-R model, meaningful work is hence seen to perform the mediation in the correlation between employee-AI collaboration and work engagement.

H4: Meaningful work mediates the relationship between Employee–AI collaboration and Work Engagement.

### 2.6 The mediating role of creative self-efficacy

According to recent research on generative AI, AI will simultaneously facilitate and limit the creative processes of employees depending on the way that people think of and use such technologies (Liang et al., 2025). These results suggest that the level of confidence of employees in their creative potentials, i.e., creative self-efficacy, may be the key factor whether AI cooperation will have positive motivating effects.

The creative self-efficacy is an area of JD-R personal resource that is domain-specific and determines the mobilization of effort and the persistence of employees toward work goals. The JD-R model assumes that the efficacy beliefs are predictors of engagement and performance in that they affect the goal setting, intensity of effort, and persistence in the presence of setbacks (Stajkovic and Luthans, 1998). Creative self-efficacy, within the context of innovation, helps to select more challenging innovation goals, devote time and attention, and stay resolute and diligent in the face of uncertainty and risk of failure, which, in turn, contributes to innovation-linked engagement and behaviors (Tierney and Farmer, 2002, 2011; Hammond et al., 2011).

As a job resource, employee-AI collaboration improves innovative self-efficacy based on mastery experiences, expansion in capabilities, and skill development, which facilitates the JD-R motivational process according to which job resources enable work engagement based on personal resources.

H5: Creative Self-efficacy mediates the relationship between Employee–AI collaboration and Work Engagement.

**Figure 1:**
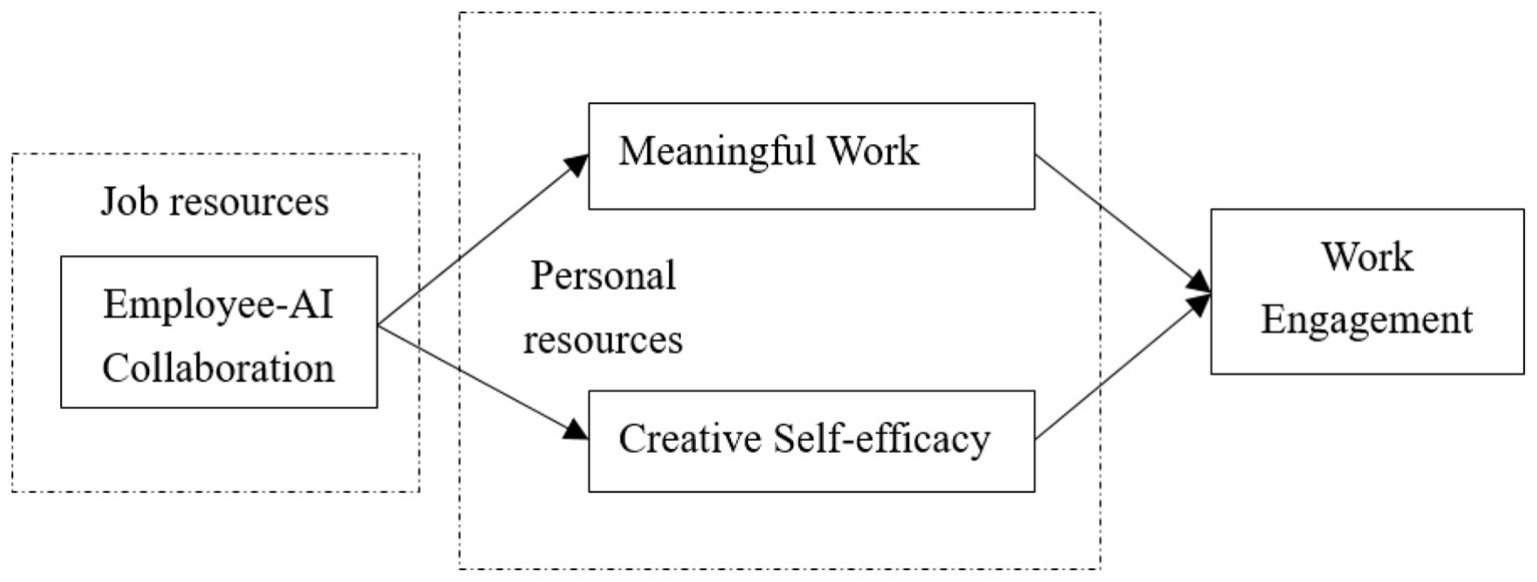
Research model.

In sum, the research model is shown below (Figure 1).

## 3 Research design

### 3.1 Sample selection and data collection

The sample of the individuals to be included in this research was majorly selected among various industries of technology firms within China, this is because China stands as a representative country due to its diverse population, high-technological environment, cultural diversity and also the researchers are likely to make substantial contributions beyond the scope of the study of human-AI interaction; In this case, data was collected among the people of China between January and April 2025. Through the snowball sampling technique, we identified employed members who participated in the study online thereby enabling them to act anonymously since this would quell their fears and anxieties. The sample data was gathered in three stages with the interviewee taking a 2 week break between stages, ensuring that the researchers reduced the likelihood of certain motives of consistency and that the questionnaires would be answered in an irresponsible fashion.

During the first stage, the participants were requested to rate employee-AI cooperation, and demographic data (gender, age, education level, etc.) was to be provided by the respondents. The total number of questionnaires issued was 700 and 623 valid responses were received hence response rate was 89. The second phase involved the participants of the first phase to fill out meaningful work scale as well as measure their creative self-efficacy with 556 responses being valid and a response rate of 89.25. The third phase required the participants to rate their work engagement, which led to 536 respondents with valid answers and a response rate of 96.4.

Incentive cash rewards of 1 yuan, 3 yuan, and 6 yuan were provided in order to promote the response rate of the questionnaires to the three rounds respectively. Those that filled the questionnaire in the earlier phase could then proceed to the questionnaire to the next stage where they would receive the required reward upon filling the questionnaire. Upon gathering the valid 536 questionnaires, we filtered the received data keenly and discovered that 20 of the questionnaires were disqualified as encompassing incomplete responses or strange patterns of responses with answers, which did not accomplish our research criterion. These samples even though they were 3.7% of the entire sample size did not largely affect our analytical results. In our opinion, it was rational to delete these samples because our calculations using G^*^ power, we determined that a sample size of 464 participants is sufficient to detect statistically significant effects. The demographics of the sample were as follows: referring to the gender composition there were 357 women (69.2) and 159 men (30.8); the results are shown in Table 1.

**Table 1:** Basic information of the sample.

**Variable**	**Options**	**Frequency**	**Percentage (%)**
Gender	Male	159	30.8
	Female	357	69.2
Age group	0-20 years	3	0.6
	21-30 years	214	41.5
	31-40 years	261	50.6
	41-50 years	27	5.2
	51 years and above	11	2.1
Highest education	Associate degree or below	35	6.8
	Bachelor's degree	350	67.8
	Master's degree	121	23.4
	Doctorate	10	1.9
Work experience	Less than 5 years	164	31.8
	6-10 years	247	47.9
	11-15 years	71	13.8
	16-20 years	8	1.6
	More than 21 years	26	5
Position level	General employee (no management responsibilities)	195	37.8
	Frontline manager (supervisor/team leader)	121	23.4
	Middle manager (department manager level)	172	33.3
	Senior manager (director and above)	28	5.4
Industry type	Information Technology/Internet/Software	145	28.1
	Finance/Banking/Insurance	37	7.2
	Manufacturing/Industrial	154	29.8
	Healthcare/Pharmaceuticals/Health	20	3.9
	Real Estate/Construction	36	7
	Professional Services (Consulting/Legal/Accounting, etc.)	32	6.2
	Other	92	17.8

### 3.2 Variable measurement

All variables in the questionnaire, except for control variables, were measured using 5-point Likert scales. To avoid language misunderstandings introduced by translation and back-translation, the English measurement scales for the variables strictly followed a “translation–back-translation” procedure, translating the items into Chinese without altering the original item semantics.

(1) Employee–AI collaboration

This study used the Employee–AI collaboration scale developed by Kong (2023) to measure the extent of employees' collaboration with AI systems. The scale contains five items and asks respondents to evaluate the degree of AI's involvement in their work processes. Example items include: “AI participates in my forecasting process.” Higher scores indicate a higher degree of collaboration between employees and AI. In this study, the scale's Cronbach's α was 0.739.

(2) Creative Self-efficacy

This study used the Creative Self-efficacy scale developed by Liu et al. (2014) to assess employees' confidence and beliefs in their own innovative capabilities. The scale includes four items, such as: “I think I am good at proposing novel ideas,” “I am confident in my ability to solve problems creatively,” “I have a knack for further supplementing and refining others' views,” and “I am good at discovering new ways to solve problems.” In this study, Cronbach's α for the scale was 0.724.

(3) Meaningful Work

This variable was measured using the scale developed by Arnold et al. (2007), with four items, such as “The work I do in this job is fulfilling.” The third item, “I do not achieve important outcomes from the work I do in this job,” was reverse-scored. In this study, the scale's Cronbach's α was 0.719.

(4) Work Engagement

This study used the Work Engagement scale developed by Schaufeli et al. (2006) to measure employees' level of engagement. The scale comprises nine items, for example, “At work, I feel full of energy.” In this study, the scale's Cronbach's α was 0.823.

(5) Control variables

This study controlled for demographic variables that may affect individual behavior and cognition, including employees' gender, age, education, work, position, section.

### 3.3 Analytical methods

This study employs hierarchical regression analysis to explore the impact of employee collaboration with artificial intelligence (AI) on work engagement. We chose hierarchical regression because it clearly demonstrates the unique contributions of each predictor variable to the dependent variable and allows for a stepwise introduction of key variables in the analysis. This method is particularly suitable for our research objectives, effectively identifying the main factors that influence work engagement. Additionally, Tabachnick and Fidell (2013) highlight in their classic statistics textbook that hierarchical regression has significant advantages in exploring variable contributions, providing important support for our choice of methodology.

## 4 Data analysis and hypothesis testing

### 4.1 Common method bias test

In this study, we employed Multi-Level Maximum Likelihood (MLMV) to analyze the data and control for potential common method bias (CMB). By evaluating the model fit indices, we found that the default model had a Comparative Fit Index (CFI) of 0.884, a Tucker-Lewis Index (TLI) of 0.870, and a Root Mean Square Error of Approximation (RMSEA) of 0.06. These indicators all show a good level of model fit. Additionally, the comparative model results indicated that after using MLMV, the path coefficients remained significant (*p* < 0.01), further confirming that no significant common method bias was detected. Therefore, it can be concluded that the research results obtained are reliable and valid.

### 4.2 Confirmatory factor analysis

To further examine discriminant validity, this study conducted confirmatory factor analysis using AMOS 26.0 on the theoretical model constructed for the study. The theoretical model was a four-factor model (Employee–AI collaboration, Creative Self-efficacy, Meaningful work, Work Engagement). Next, three competing models were constructed: a three-factor model (Employee–AI collaboration, Creative Self-efficacy + Meaningful work, Work Engagement), a two-factor model (Employee–AI collaboration, Creative Self-efficacy + Meaningful work + Work Engagement), and a single-factor model (Employee–AI collaboration + Creative Self-efficacy + Meaningful work + Work Engagement). Model fit indices are shown in Table 2. The results show that the four-factor model has the best goodness of fit and is clearly superior to the other competing models, This further establishes effective differences among the variables while indicating that the overall model fit is satisfactory.

**Table 2:** Results of Confirmatory Factor Analysis (CFA).

**Model**	**χ^2^**	**df**	**χ^2^/df**	**RMSEA**	**RMR**	**IFI**	**GFI**	**CFI**
Four-factor model	518.794	203	2.556	0.055	0.023	0.903	0.915	0.902
Three-factor model	722.226	206	3.506	0.07	0.025	0.841	0.879	0.84
Two-factor model	844.421	208	4.06	0.077	0.027	0.804	0.864	0.803
Single-factor model	1093.059	209	5.23	0.091	0.035	0.728	0.823	0.726

### 4.3 Descriptive statistics and correlations

Table 3 presents the correlation analysis results among the primary study variables (N = 516). The analysis results indicate that the association between EAIC and WM is significant and positive. (r = 0.360, *p* < 0.01), with CSE (r = 0.350, *p* < 0.01), and with WE (r = 0.431, *p* < 0.01). The results indicate a significant and positive relationship between WM and CSE. (r = 0.434, *p* < 0.01) and with WE (r = 0.568, *p* < 0.01). CSE is also significantly and positively correlated with WE (r = 0.530, *p* < 0.01). All correlation coefficients are statistically significant and in the expected directions, ranging from 0.350 to 0.568, indicating that there are no multicollinearity concerns in subsequent analyses.

**Table 3:** Results of descriptive statistical analysis (*N* = 516).

	**M**	**SD**	**Gender**	**Age**	**Edu**	**Work**	**Position**	**Section**	**EAIC**	**MW**	**CSE**	**WE**
Gender	1.69	0.46	1									
Age	2.67	0.68	0.094^*^	1								
Edu	2.21	0.58	0.012	0.128^**^	1							
Work	2	0.99	0.069	0.802^**^	−0.011	1						
Position	2.06	0.96	0.110^*^	0.464^**^	0.247^**^	0.425^**^	1					
Section	3.44	2.18	0.032	0.077	−0.155^**^	0.161^**^	−0.036	1				
EAIC	4.23	0.51	−0.090^*^	0.038	0.109^*^	0.001	0.110^*^	−0.195^**^	1			
MW	4.29	0.45	−0.083	0.137^**^	0.082	0.124^**^	0.179^**^	−0.184^**^	0.360^**^	1		
CSE	4.2	0.51	−0.033	0.024	0.049	−0.005	0.120^**^	−0.185^**^	0.350^**^	0.434^**^	1	
WE	4.21	0.46	0.028	0.196^**^	0.095^*^	0.177^**^	0.218^**^	−0.154^**^	0.431^**^	0.568^**^	0.530^**^	1

Notes: ^**^p < 0.01, ^*^p < 0.05; EAIC, Employee–AI collaboration; WM, Meaningful work; CSE, Creative Self-efficacy; WE, Work Engagement.

### 4.4 Hypothesis testing

Based on the stepwise linear regression results in Table 4, the tests of the five hypotheses are as follows: EAIC has a significant positive effect on WE(β = 0.365, *p* < 0.001; Model 5), supporting Hypothesis 1;EAIC has a significant positive effect on WM (β = 0.28, *p* < 0.01; Model 2), supporting Hypothesis 2; it explains 17.6% of the variance in WM. Employee–AI collaboration has a significant positive effect on CSE (β = 0.317, *p* < 0.001; Model 4), supporting Hypothesis 3; it explains 14.4% of the variance in CSE. Hypothesis 4 was supported: WM partially mediates the relationship between Employee–AI collaboration and WE. After adding WM (Model 6), it had a significant positive effect on WE (β = 0.461, *p* < 0.001), while the direct effect of Employee–AI collaboration decreased from 0.365 to 0.236 but remained significant; the model's explanatory power increased from 24.0% to 40.8% (Δ*R*^2^ = 0.168, *p* < 0.001). Hypothesis 5 was supported: CSE partially mediates the relationship between Employee–AI collaboration and WE. After adding CSE (Model 7), it had a significant positive effect on WE (β = 0.381, *p* < 0.001), while the direct effect of Employee–AI collaboration decreased from 0.365 to 0.244 but remained significant; the model's explanatory power increased significantly (Δ*R*^2^ =0.152, *p* < 0.001). All models' F-values were significant (*p* < 0.001), indicating good overall model fit.

**Table 4:** Stepwise linear regression results.

**Variables**		**MW**		**CSE**		**WE**		
		**M1**	**M2**	**M3**	**M4**	**M5**	**M6**	**M7**
Controls	Gender	−0.097^*^	−0.067^*^	−0.044	−0.01	0.044^*^	0.075^*^	0.048
	Age	0.025^*^	0.018	0.011	0.004	0.038^*^	0.03	0.037
	Edu	0.014	0.001	−0.011	−0.026	0.006^*^	0.005^*^	0.015
	Work	0.035	0.039^*^	−0.022^*^	−0.017	0.0488^*^	0.03^*^	0.054^*^
	Position	0.06^*^	0.045^*^	0.07	0.053^*^	0.044^*^	0.024	0.024^*^
	Section	−0.039	−0.027	−0.041^*^	−0.028	−0.02	−0.007	−0.009
Independent variable	EAIC		0.28^**^		0.317^***^	0.365^***^	0.236^***^	0.244^***^
Mediators	MW						0.461^***^	
	CSE							0.381^***^
	*R*	0.287	0.420	0.223	0.380	0.490	0.639	0.626
	*R* ^2^	0.082	0.176	0.05	0.144	0.24	0.408	0.392
	Δ*R*^2^	0.082	0.094	0.05	0.095	0.153	0.168	0.152
	*F*	7.609^***^	15.518^***^	4.431^***^	12.242^***^	22.93^***^	43.626^***^	40.889^***^

Notes: ^***^p < 0.001, ^**^p < 0.01, ^*^p < 0.05; EAIC, Employee—AI collaboration; MW, Meaningful work; CSE, Creative Self-efficacy; WE, Work Engagement.

Based on the above regression results, to further verify the robustness of the mediation effects, this study used the SPSS PROCESS macro (Model 4) developed by (Hayes 2013) to test mediation, employing a bootstrap resampling method (5,000 samples, 95% confidence intervals) to assess the significance of indirect effects. The results are shown in Tables 5, 6: Table 5 (mediator: meaningful work) shows that the total effect of employee-AI collaboration on WE is 0.3897 [95% CI = (0.319, 0.4603)], the direct effect is 0.2351 [95% CI = (0.1691, 0.3012)], and the indirect effect via WM is 0.1545 [95% CI = (0.0943, 0.2437)]. Because the confidence interval of the indirect effect does not include 0, the mediation effect of WM is significant.

**Table 5:** Decomposition of the total, direct, and indirect effects of meaningful work.

	**Coeff**	**BootSE**	**BootLLCI**	**BootULCI**
Total effect	0.3897	0.036	0.319	0.4603
Direct effect	0.2351	0.0336	0.1691	0.3012
Indirect effect	0.1545	0.0381	0.0943	0.2437

**Table 6:** Decomposition of the total, direct, and indirect effects of creative self-efficacy.

	**Coeff**	**BootSE**	**BootLLCI**	**BootULCI**
Total effect	0.3897	0.036	0.319	0.4603
Direct effect	0.2531	0.0343	0.1857	0.3206
Indirect effect	0.1365	0.038	0.0771	0.2234

Table 6 (mediator: creative self-efficacy) shows that the total effect of Employee-AI collaboration on WE is 0.3897 [95% CI = (0.319, 0.4603)], the direct effect is 0.2531 [95% CI = (0.1857, 0.3206)], and the indirect effect via CSE is 0.1365 [95% CI = (0.0771, 0.2234)]. Because the confidence interval of the indirect effect does not include 0, the mediation effect of CSE is significant. The bootstrap results further confirm Hypotheses 3 and 4, namely that WM and CSE both play significant partial mediating roles in the relationship between Employee-AI collaboration and WE.

**Figure 2:**
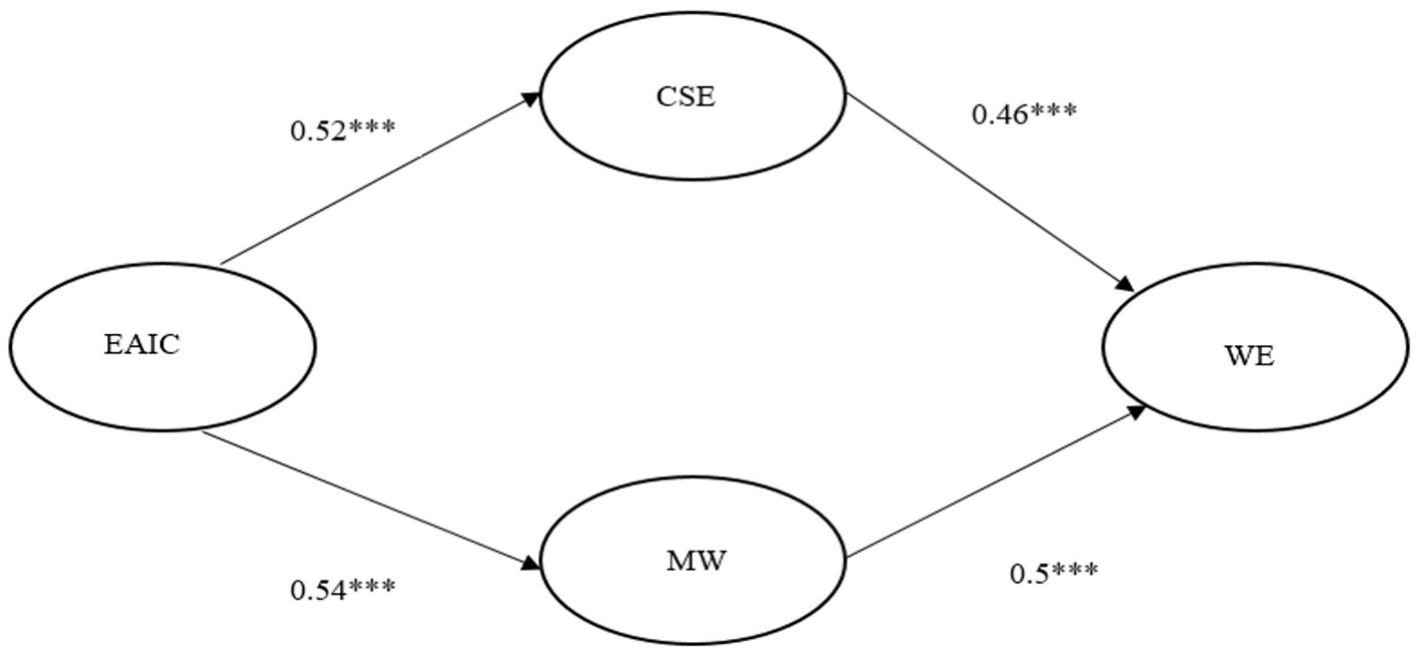
SEM path coefficient diagram. Notes: ***p < 0.001; EAIC, Employee–AI collaboration; MW, Meaningful work; CSE, Creative Self-efficacy; WE, Work Engagement.

To further validate the mediating effects of innovative self-efficacy and the sense of meaningful work between EAIC and WE, this study applied SEM analysis and used Amos 26.0 software to establish a structural equation model to test the mediating roles of CSE and MW between EAIC and WE. The results indicated that EAIC significantly positively influenced MW (β = 0.54, *p* < 0.001), and MW significantly positively influenced WE (β = 0.5, *p* < 0.001), supporting Hypothesis 4 once again. EAIC also significantly positively influenced CSE (β = 0.52, *p* < 0.001), and CSE significantly negatively influenced WE (β = −0.46, *p* < 0.001), thus providing support for Hypothesis 5 again. The standardized path model is shown in Figure 2.

## 5 Discussion

This study explores how employee–AI collaboration affects work engagement and examines the mediating roles of meaningful work and creative self-efficacy. The core findings indicate that Hypothesis 1 is supported: employee–AI collaboration significantly enhances work engagement (β = 0.365, *p* < 0.001). The testing of Hypothesis 4 shows that meaningful work plays an important partial mediating role in this relationship. This means that when employees perceive their work as meaningful, they are more likely to exhibit higher levels of work engagement when engaging in employee–AI collaboration. This finding aligns with Deci and Ryan (2000) self-determination theory, which suggests that intrinsic motivation (such as a sense of meaning in work) can stimulate employees' active participation. Additionally, Hypothesis 5 is also supported, with creative self-efficacy acting as another important partial mediating variable. This suggests that employees' confidence in their abilities contributes to enhanced engagement during employee–AI collaboration, which is consistent with the research by Tierney and Farmer (2002), emphasizing the role of creative self-efficacy in driving employee motivation.

Through the analysis of these two mediating variables, we can see their mechanisms of influence on employee work engagement. First, meaningful work not only increases employees' work positivity but also enhances their sense of engagement, making them feel more fulfilled and satisfied when participating in employee–AI collaboration. This psychological state can promote innovation in employees‘ work, further boosting work engagement. Second, creative self-efficacy encourages employees to explore and utilize AI technologies, thereby enhancing their sense of self-efficacy. This increase in self-efficacy can effectively motivate employees, prompting them to actively participate in their work and improve overall job performance.

The results of this study share similarities with the research conducted by Haesevoets et al. (2021), which also emphasizes the positive impact of employee–AI collaboration on employee work outcomes. However, our research further deepens this discussion, particularly in terms of psychological variables, highlighting how meaningful work and creative self-efficacy play key roles in this process.

## 6 Theoretical contributions

Increasing the knowledge on human-AI teamwork. In response to the demands of scholars to learn more about the psychological processes of human-AI collaboration (Wilson and Daugherty, 2018; Jarrahi, 2018), we combine four dimensions information exchange, division of tasks, mutual adaptation and collaboration outcomes to form a multidimensional conceptual framework of employee-AI collaboration, which will provide more detailed and versatile measuring instruments in further studies.

This theory transcends technology acceptance or frequency of use by conceptualizing employee-AI work as an embedded job resource that influences the motivation in employees, instead of an adoption decision or interaction problem as in technology acceptance and human-computer interaction views.

More to the point, the present research reveals the phenomenon of the human-AI collaboration augmentation (Raisch and Krakowski, 2021). The USP of the given research is that it will thoroughly explore the way the collaboration between employees and AI may revolutionize the traditional interactions in the workplace. The fact that our study isolates certain psychological mechanisms through which the engagement occurred in the instance of Meaningful work and Creative Self-efficacy, demonstrates not only the new findings themselves, but the framework upon which organizations can better engage their employees by implementing strategic AI adoption. This factor has been comparatively under researched in the current literature, and our results are therefore useful in this regard.

## 7 Managerial implications

In order to successfully utilize employee-AI collaboration (E-AIC) as the resource that allows promoting work engagement, organizations may introduce some specific tools that should be geared toward declaring the personal resources of Meaningful work and Creative Self-efficacy. These include:

Training Programs: Establish elaborate training programs to train employees on how to co-operate effectively with AI tools. This training must be considered on both technical abilities one will need to use AI technologies, and soft skills that facilitate teamwork, e.g., communication and collaboration. The personal confidence of employees (employee empowerment) can be strengthened by providing knowledge and skill enhancement that would help them enhance their Creative Self-efficacy and general competence in the workplace.

Feedback Mechanisms: One time processes can be established through which employees can exchange their experience and perspectives on AI collaboration. By using reports and focus groups as a means of collecting this data, the management would be able to adjust AI tools to the needs of the employees in better ways. This procedure in addition to boosting the F datum of a Meaningful work, also makes it a culture of open communication, which could substantially raise employee involvement.

Recognition Programs: Introduce recognition programs which could celebrate the successful collaboration between the employees and the AI. Rewarding those employees who creatively utilize AI tools to streamline their work can increase their feeling of achievement, which, in turn, increases their Creative Self-efficacy and solidifies beneficial collaborative performance.

Cross-Functional Teams: Develop cross-functional teams, which would involve a variety of skills and views to undertake work concerning the field of AI. Meaningful work is enhanced because these groups enable workers to view the effects of their work in more than one perspective and this gives rise to the collaborative culture and encourages innovation.

## 8 Limitations of the research and future prospects

In spite of the fact that this research sheds valuable light on the processes according to which employee-AI cooperation produces the desired results, it contains numerous limitations as well, which also lead to new opportunities in the future studies.

The first one is that the sample is predominantly comprised of knowledge workers in China and therefore, has a relatively homogenous cultural background that could restrict cross-cultural generalizability. The attitudes of the employees to human-AI collaboration and their experience with it might vary depending on the cultural context. Future studies have to confirm the results in new cultural and industry contexts and investigate the moderating date of cultural values and industry characteristics.

Second, the scale employed to conduct research in this study has been developed by Kong (2023) and it is mostly aimed at people working in occupations related to proteins. Because our research failed to consider the occupational features of the non-protein employees, this can restrain the applicability of the scale in a variety of occupational situations. Thus, the future studies need to examine the appropriateness of this scale to employees of other categories and how well it can be used to evaluate employee-AI cooperation.

Third, even though meaningful work can conceivably be able to promote creative self-efficacy through reinforcing the sense of purpose in employees and helping them feel that the contributions they make are valuable, the study of serial mediation effects was not within the scope of the current study and it presents a promising research topic in the future.

Lastly, the paper largely uses self-report measures and therefore subjective bias can also be involved. Simultaneously, the dynamic formation of the human-AI collaborative relationships can be poorly reflected in a fixed research design. Future studies ought to incorporate both objective behavioral measures and other-reports so as to create a more holistic system of measurement and adhere to techniques like experience sampling in order to analyze the dynamic nature of collaboration experience.

## 9 Conclusions

In line with the ongoing data showing that the cooperation of employees with AI may produce both beneficial and detrimental behavioral effects (Kellogg et al., 2020; Meng et al., 2025; Sun et al., 2025), the presented findings additionally show that this type of cooperation can moderately affect the motivation of employees based on specific psychological resource processes, which is proven by the high quality of empirical design, as well as, identifies the mediating effects of meaningful work and creative self-efficacy. Such results do not only add value to the theoretical framework of human-AI collaboration and work engagement but also offer scientific directions to the human resource management practices in the era of AI. The findings indicate that the usefulness of AI technology is not only in enhancing efficiency, but also providing psychological resources and work experience to the employees due to the successful interaction of humans and AI. The given insight also has significant implications on how to establish positive human-AI collaborative relationships and harmonious development between technology and human work. Nevertheless, despite some shortcomings, this paper offers a significant theoretical background and practice basis in the exploration of human-AI cooperation during the AI age, which future studies can be further elaborated and extended.

## Data Availability

The raw data supporting the conclusions of this article will be made available by the authors, without undue reservation.

## Appendix

**Table A1:** Appendix A: Scale Items.

**EAIC**	**EAC1. AI participates in my decision-making process**.
	EAC2. AI participates in my prediction process.
	EAC3. AI participates in my problem-solving process.
	EAC4. AI participates in my information identification and evaluation process.
	EAC5. AI participates in my problems, opportunities, or risk recognition process.
CSE	CSE1. I believe I excel at generating novel ideas.
	CSE2. I have confidence in my ability to creatively solve problems.
	CSE3. I have a knack for further refining others' viewpoints.
	CSE4. I am skilled at discovering new ways to solve problems.
WM	MW1:The work I do in this job is fulfilling.
	MW2:The work I do in this job is rewarding
	MW3:I do not achieve important outcomes from the work I do in this job. (reverse-scored)
	MW4:I am able to achieve important outcomes from the work I do in this job.
WE	WE1:At my work, I feel bursting with energy.
	WE2:At my job, I feel strong and vigorous.
	WE3:When I get up in the morning, I feel like going to work.
	WE4:I am enthusiastic about my job.
	WE5:My job inspires me.
	WE6:I am proud of the work that I do.
	WE7:I am immersed in my work.
	WE8:I get carried away when I'm working.
	WE9:I feel happy when I am working intensely.
